# The Potassium-Uptake Systems, Trk and Kdp, Coordinately Contribute to Growth Regulation and Survival of *M. tuberculosis* in Ion-Depleted and Acidic Environments

**DOI:** 10.3390/ijms27093962

**Published:** 2026-04-29

**Authors:** Ayman G. E. Osman, Maborwa T. Matjokotja, Mushal Allam, Arshad Ismail, Ronald Anderson, Moloko C. Cholo

**Affiliations:** 1 Department of Immunology, Faculty of Health Sciences, University of Pretoria, Pretoria 0001, South Africa; 2 Sequencing Core Facility, National Institute for Communicable Diseases, Division of the National Health Laboratory Service, Johannesburg 2192, South Africa; 3 Department of Biochemistry and Microbiology, Faculty of Science, Engineering and Agriculture, University of Venda, Thohoyandou 0950, South Africa

**Keywords:** *Mycobacterium tuberculosis*, K^+^-uptake systems, Trk and Kdp, growth, survival, planktonic cultures, biofilm formation, macrophages

## Abstract

The *Mycobacterium tuberculosis* bacterium encodes two active potassium (K^+^)-uptake transport systems, the Trk and the Kdp. The Trk is the low-affinity K^+^ transporter, consisting of two TrkA proteins, while the Kdp consists of the high-affinity K^+^ transporter KdpFABC and the two-component system KdpDE. Both transporters are utilised by the bacteria for growth and survival. During growth, the bacteria utilise the constitutively expressed Trk and suppress the Kdp, but upregulate both transporters during survival. In the current study, we investigated the interactive effects of these systems on bacterial growth and survival. This was achieved by first constructing a *M. tuberculosis* mutant strain in which both the Trk and Kdp systems were inactivated by homologous recombination. The mutant was evaluated for its growth kinetics in planktonic cultures, as well as survival in biofilm and macrophage cultures. The constructed *M. tuberculosis* mutant showed faster growth rates in planktonic cultures, but was attenuated for both biofilm formation and intracellular survival in isolated human monocyte-derived macrophages. These results illustrate that both K^+^-uptake systems are essential to sustain slow rates of bacterial growth, as well as for bacterial persistence in hostile environments via optimisation of biofilm formation, and intracellular survival in macrophages. (Words: 194)

## 1. Introduction

Potassium (K^+^) is a major cellular cation typically maintained at higher concentrations inside cells than in the extracellular milieu. It is utilised by bacteria for intracellular pH homeostasis to near-neutral levels (pH of 7.4 to 7.8) [1,2,3]. The elevated K^+^ and pH levels contribute to various cellular functions such as regulation of membrane potential, enzyme activity, protein synthesis and osmotic pressure [4,5,6]. Most bacteria maintain monovalent cation homeostasis in varying external K^+^ and pH conditions, enabling growth and survival. *M. tuberculosis* bacteria grow optimally in environments of elevated extracellular K^+^ and pH, while transitioning to dormancy for survival in low-K^+^ [4,6,7] and acidic pH environments [8,9,10,11].

*M. tuberculosis* possesses two active, K^+^-uptake transport systems, namely the Trk and Kdp [12,13,14]. The Trk is the low-affinity K^+^-uptake transporter, consisting of two highly homologous TrkA proteins, CeoB and CeoC [12]. Both proteins contain nicotinamide adenine dinucleotide (NAD^+^)-binding motifs, which serve as binding sites for K^+^ and hydrogen (H^+^) ions [15]. The Kdp system consists of the high-affinity phosphorylated (P)-type adenosine triphosphatase (ATPase) K^+^-uptake transporter, KdpFABC, consisting of the K^+^-transporting KdpA, P-type ATPase KdpB, and regulatory KdpF and KdpC subunits [16], as well as the two-component system (TCS) KdpDE, consisting of the histidine kinase KdpD and response regulator KdpE [17,18,19]. The KdpDE senses adverse environmental signals such as low K^+^ [4,16,17], acidic pH [20,21], nutrient starvation [22] and hypoxia [23], leading to activation of the *kdpFABC* operon.

The two K^+^ transporters have been shown to function during bacterial growth and survival. We have previously shown that during growth, the *M. tuberculosis* bacteria preferentially utilise the constitutively expressed Trk as the main active K^+^-uptake transporter, using single Trk (CeoBC)-deletion mutant strains [8,13]. This contention is supported by observations in *Mycobacterium smegmatis*, in which the TrkA system was shown to be responsible for bacterial growth, membrane potential and pH modulation [24]. In the case of the KdpFABC, this system is suppressed during bacterial growth [8,13]. However, in our aforementioned earlier studies, we showed that this system is induced in the absence of the Trk, resulting in increased K^+^ uptake, as well as growth rates, revealing its ability to function during growth as a backup in high-K^+^ environments [8,13].

In the case of *M. tuberculosis*, both the Trk and Kdp transporters are operative during *M. tuberculosis* bacterial survival. In the context of the Trk system, the CeoB protein has been shown to be upregulated in low-K^+^ environments such as biofilm-forming cultures [25,26] while both *ceoB* and *ceoC* genes were shown to be upregulated in the late acidic phases of planktonic cultures [8]. In the case of the KdpFABC transporter, the *kdpA*, *kdpB* and *kdpC* genes were upregulated in K^+^-limiting conditions [4,5,27], while the entire *kdpFABC* operon was induced in acidic planktonic conditions [8,21]. In other mycobacterial species, such as *Mycobacterium marinum*, the *kdpA* was also found to be induced under low-K^+^ conditions, also playing a role in pH homeostasis [28].

Importantly, the functions of the Trk and Kdp systems during varying growth conditions have been shown to be regulated by the KdpDE system. In the absence of the KdpDE system, the genes encoding these two transporters during growth were dysregulated with respect to their involvement in regulating K^+^ uptake and growth rates [21]. Other previous studies have also reported on the interactions of the KdpD and KdpE [29], as well as on the upregulation of the *kdpE* gene during bacterial growth [30]. Similar to involvement in bacterial growth, the KdpDE system has also been implicated in bacterial survival, as shown by the upregulation of both the *kdpD* and *kdpE* genes in K^+^-limiting [4,17,18,23,31] and acidic conditions in planktonic cultures [8,21].

In vivo, *M. tuberculosis* bacteria are able to grow and survive intracellularly in macrophages, and also survive extracellularly in biofilm and granuloma lesions [28,32]. The Trk and the KdpDE systems have been shown to be required for bacterial survival in macrophages, as shown by the upregulation of the *ceoB* [10,33] and the *kdpE* [34] genes in the late phase of post-bacterial infection in macrophages. However, although not yet described for the KdpFABC system of *M. tuberculosis*, the *kdpF* gene of *M. bovis*, which has high genomic sequence similarity to that of *M. tuberculosis*, has been shown to be required for bacterial intracellular survival in a murine model of experimental infection, as well as in human monocyte-derived macrophages [27], highlighting the potential role of the *M. tuberculosis* KdpFABC transporter in intracellular bacterial survival.

However, based on the essentiality of each of the Trk and Kdp systems during bacterial growth and survival, the current study was designed to investigate cooperation between these mycobacterial K^+^ transporters during various growth conditions. These objectives were achieved by first constructing a mutant strain of *M. tuberculosis* in which both the Trk and Kdp (KdpDE and KdpFABC) systems were inactivated using homologous recombination. The mutant was thereafter evaluated for its growth kinetics in planktonic cultures and survival in biofilm and macrophage cultures by comparing its responses to those of the wild-type (WT) strain.

## 2. Results

### 2.1. Construction of the Kdp-Trk (KT)-Triple-Gene Mutant Construction of M. tuberculosis

**Figure 1:**
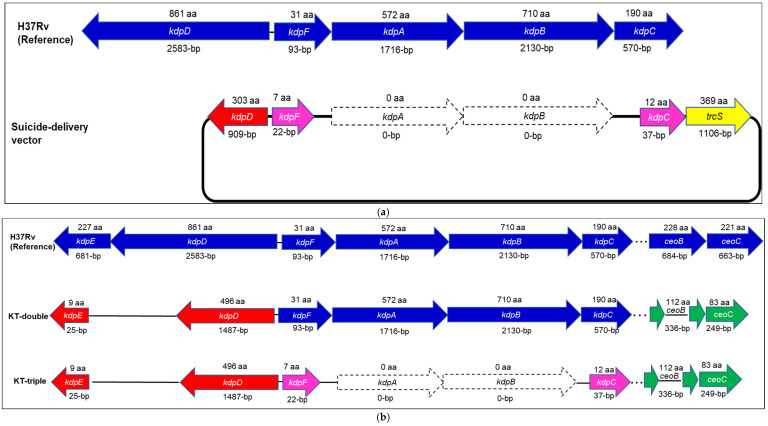
Schematic illustration of the construction of the (**a**) suicide delivery vector (SDV), *pRB5kdpDFC17′*, showing genomic (bp: base-pair) and amino acid (aa) sequences of the *kdpFABC* genes, and (**b**) the Kdp-Trk (KT: Δ*kdpDE*-Δ*kdpFABC*-Δ*ceoBC*)-triple-gene knockout mutant by homologous recombination showing genomic sequences of the *kdp* and *trk* genes in the wild-type (WT), Kdp-Trk (KT: Δ*kdpDE*- Δ*ceoBC*)-double- and KT-triple-knockout mutant strains of *M. tuberculosis.* The colours represents the following: Blue, intact WT genes; Red, mutated *kdpDE* genes; Green, mutated *ceoBC* genes, Pink, mutated and trancated *kdpF* and *kdpC*; white, complete deletion of *kdpA* and *kdpB* genes. The symbols represent the following: The black rectangle in Figure 1a, plasmid; Dotted line, genes are located at distal positions from each other; black lines between genes, deleted parts of gene/s.

The Trk system consists of two TrkA proteins, CeoB and CeoC, encoded by the *ceoBC* operon, while the Kdp system consists of the KdpFABC and the KdpDE systems encoded by the respective *kdpFABC* and *kdpDE* operons [12]. The mutant construction was achieved by inactivation of the *kdpFABC* operon of the KdpDE-Trk (KT)-double-knockout mutant strain, which carries Δ*kdpDE* and Δ*ceoBC::hyg^R^* mutations (Table 1) [21] using homologous recombination. This involved first the construction of the suicide-delivery vector (SDV), which was used for the development of the single cross-over (SCO) clones, followed by generation of the double cross-overs (DCOs) [35]. The resultant mutant was the *M. tuberculosis* Kdp-Trk (KT)-triple-gene knockout mutant, characterised by mutations of these three operons, viz, *ceoBC*, *kdpFABC* and *kdpDE* (Figure 1).

#### 2.1.1. The SDV Construct

The SDV was constructed following the ligase-independent cloning (LIC) procedure, as previously described [36]. The plasmid was achieved by sequential insertion of the 1164-bp *kdpDF’*- and 1143-bp *kdpC′*-polymerase chain reaction (PCR)-synthesised inserts at the BsaI and BseRI LIC sites of the pNILRB5 vector, respectively (Figure 1a, Supplementary Figure S1a). The resultant vector was the *pRB5kdpDFC17′* plasmid, carrying the mutated *kdpDFC’* allele.

Only four blue, Kan^R^, Suc^R^ *Escherichia coli* colonies were isolated, and no other colonies with different phenotypes were detected. The SDV construction from the four colonies was genotypically confirmed by PCR analysis showing amplification of the PCR-synthesised fragments (Figure 1a, Supplementary Figure S1a). Only one clone was used for whole-genome sequencing (WGS) analysis, showing the presence of the truncated *kdpDF′* and *kdpC*′ reads and the deletion of the *kdpA* and *kdpB* genes at the *M. tuberculosis kdpFABC* operon (Figure 1a, Supplementary Figure S1b).

#### 2.1.2. The KT-Triple-Gene Knockout

The mutant construction was achieved by replacing the WT *kdpFABC* operon in the KT (Δ*kdpDE*-Δ*ceoBC::hyg^R^*)-double-gene-knockout strain [21] (Table 1) with the mutated Δ*kdpDFC* by homologous recombination. This resulted in deletion of an approximately 4450 bp fragment of the 4509 bp *kdpFABC* operon. The remaining 59 bp gene fragment consisted of the 22 bp *kdpF* (93 bp original) and 37 bp *kdpC* (570-bp original) genes. The resultant mutant strain was the KT (Δ*kdpDE*-Δ*kdpFABC*-Δ*ceoBC::hyg^R^*)-triple-gene knockout.

The mutant was characterised phenotypically by the isolation of small, white Kan^S^, Suc^R^ colonies on a 7H10 agar plate. The mutant clones were confirmed genotypically by PCR analysis, showing an amplification of only the 868 bp Δ*kdpDFC* fragment (Figure 1b, Supplementary Figure S2a), and by WGS analysis (Figure 1b, Supplementary Figure S2b), showing the absence of reads at the *kdpFABC* genes. Further sequencing analysis confirmed mutations at the *kdpDE* and *ceoBC* operons.

### 2.2. Inoculum

The inoculum sizes of the WT and the KT-triple-gene knockout mutant strains were determined by optical density (OD) measurements to a reading of 1.2 at 540 nm. The OD readings yielded 4.4 × 10^4^ ± 3.4 × 10^4^ and 1.57 × 10^5^ ± 1.15 × 10^5^ colony-forming units (cfu)/mL for the WT and the mutant strains, respectively. The mutant strain was diluted 4× to yield 3.95 × 10^4^ cfu/mL.

### 2.3. Planktonic Growth Analysis

#### 2.3.1. Rates of Planktonic Growth

**Figure 2:**
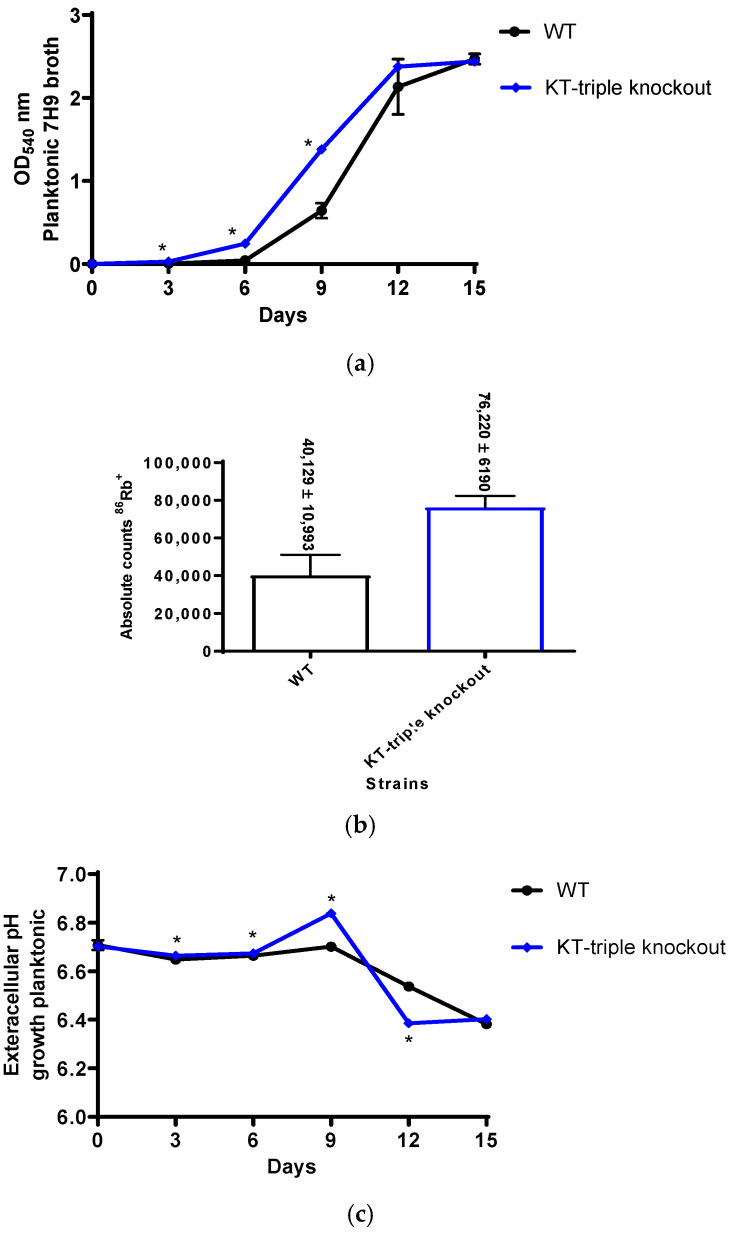
Assays of bacterial growth of the wild-type (WT) and the Kdp-Trk (KT)-triple-gene knockout mutant strains performed in 7H9 broth medium. The results are a minimum of three experiments performed in duplicate. Statistical differences at *p* values ≤ 0.05 are represented by *, representing a comparison of responses between the WT strain and the mutant strain. The results show (**a**) rates of bacterial growth expressed as optical density (OD) measurements at different time points (Supplementary Table S1). The *p* values were all 0.0022 for day (D)3, D6 and D9, while they were 0.0649 and 0.59 for D12 and D15, respectively; (**b**) rubidium (^86^Rb^+^) uptake, represented as absolute cpm with the achievable *p* value of 0.0022; and (**c**) extracellular pH levels represented by the absolute values at various time points (Supplementary Table S2). The *p* values were 0.0163, 0.0104, 0.0022, 0.0022 and 0.0304 for D3, D6, D9, D12 and D15, respectively.

To investigate the role of K^+^-uptake transporters in metabolic growth, the KT-triple-knockout mutant strain was analysed for growth rates during the logarithmic phase under planktonic conditions. Planktonic growth was monitored by measuring the OD of the cultures at 540 nm. During the logarithmic phase, growth based on OD measurements is categorised into early-log (OD = 0.1–0.3), mid-log (OD = 0.4–0.6) and late-log phases (OD = 2.0–2.3). The rates of bacterial growth were determined by measurements of bacterial cultures at consecutive three-day time points (Figure 2a; Supplementary Table S1).

Each strain attained the early-log at D6, while the mutant achieved mid-log and late-log phases earlier than the WT strain, which reached these phases on D9 and D12, respectively. Despite reaching the early phase at the same time point, the mutant exhibited higher growth than the WT (*p* < 0.05), indicating faster growth rates across all three growth phases. However, during the late-log phase, although the difference was not statistically significant (D15, *p* value = 0.59), the WT grew faster than the mutant strain.

#### 2.3.2. Rubidium (^86^Rb^+^)-Uptake Efficiency

To determine the effect of K^+^-uptake efficiency on bacterial growth rates, K^+^-uptake efficiencies of the WT and the KT-triple-gene knockout mutant strains were evaluated for their K^+^-uptake efficiencies using uptake of rubidium-86 (^86^Rb^+^) radioisotope as a surrogate, and the results are represented by absolute counts per minute (cpm), as previously described [7,8,13,21]. The absolute cpm of the mutant strain were significantly higher than those of the WT strain (*p* value = 0.0022) (Figure 2b), showing higher K^+^-uptake efficiency by the mutant than the WT strain.

#### 2.3.3. Changes in Extracellular pH Levels During Planktonic Growth

To evaluate the effect of extracellular pH on bacterial growth rates, the extracellular pH measurements were determined in undiluted supernatant fractions of planktonic cultures used for rates of growth determination, as absolute pH values, at the different time points, as previously described [21].

The pH levels were 6.7 ± 0.02 at D0 for both strains. The pH levels increased at early-log (D6) and reaching optimum levels at the mid-log phase (D9) while decreasing to acidic levels at the late-log phase (D12) (Figure 2c; Supplementary Table S2).

In relation to the WT strain (Figure 2c; Supplementary Table S2), the pH levels in the mutant culture supernatants were significantly more alkalinic than those of the WT at early- (D6, *p* value = 0.01) and mid-log (D9, *p* value = 0.0022) phases, corresponding to the faster growth rates of the mutant at these time points. Interestingly, at the late-log phase, the extracellular pH values of the mutant were more acidic than those of the WT (D12, *p* value = 0.0022), resulting in attenuation of bacterial growth rates.

In summary, the results for bacterial growth showed that during planktonic growth, the mutant strain demonstrated faster rates of growth than the WT strain. This corresponded with higher K^+^-uptake efficiencies of the mutant strain and elevated external pH, both of which support bacterial growth [1,8,13]. However, at the late-log phase, the mutant attained more sluggish bacterial growth, due to more acidic extracellular environments, known to attenuate bacterial K^+^ uptake [2,8,9].

### 2.4. Biofilm Growth Analysis

#### 2.4.1. Rates of Biofilm Formation

To investigate the role of K^+^-uptake transporters in bacterial survival in biofilm environments, the mutant strain was evaluated for its biofilm formation. The biofilm formation of the WT and the KT-triple-knockout mutant strains was determined by quantitating the amounts of biofilm biomass in the cultures, using a crystal violet (CV)-based staining procedure at OD 570 nm over five weeks [37,38,39,40]. The rates of biofilm formation by each strain were determined by measuring biofilm growth at W1, W3 and W5 time points, corresponding to the early-, mid- and late-biofilm phases.

**Figure 3:**
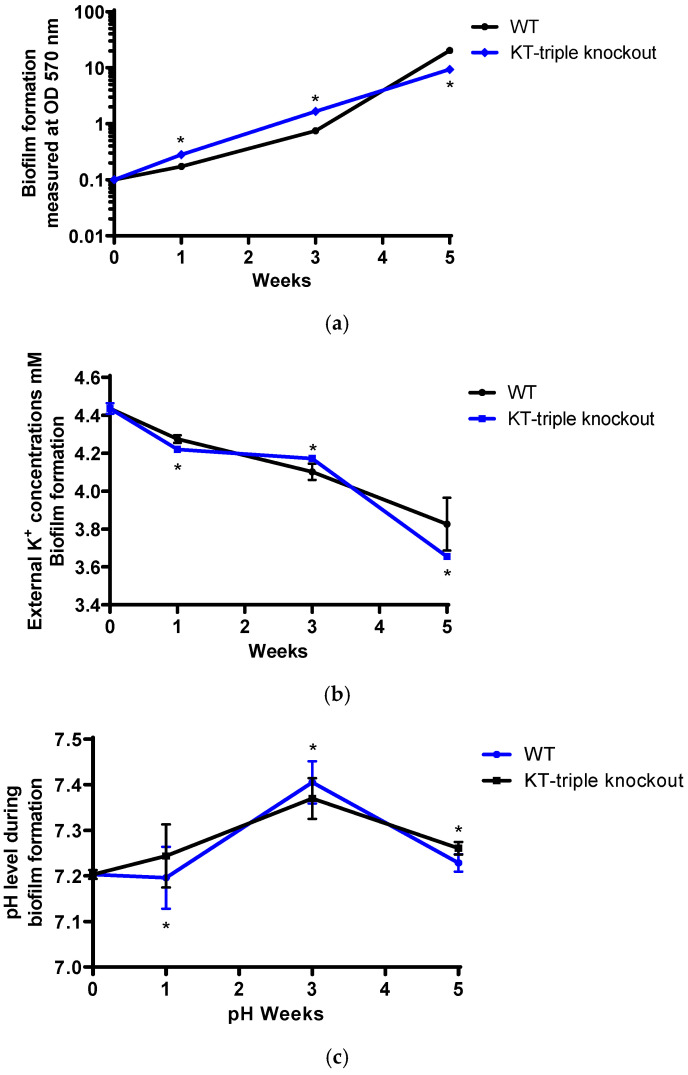
Biofilm formation by the wild-type (WT) and the Kdp-Trk (KT)-triple-gene knockout mutant strains performed in Sauton’s broth medium over five weeks. The results are a minimum of three experiments performed in duplicate. Statistical differences at *p* values ≤ 0.05 are represented by *, representing a comparison of responses between the WT strain and the mutant strain. The results show (**a**) the rates of biofilm formation of the WT and mutant strains (Supplementary Table S3), showing *p* values of 0.0022 at all time points; (**b**) the extracellular potassium (K^+^) concentrations (mM) (Supplementary Table S4), showing the *p* values of 0.0022, 0.0082 and 0.016 for weeks (W)1, W3 and W5, respectively; (**c**) extracellular pH levels (Supplementary Table S5), showing the *p* values of 0.0022, 0.0022 and 0.0022 for W1, W3 and W5, respectively.

Both strains formed biofilm at the three time points (W1, W3 and W5) (Figure 3a, Supplementary Table S3). The biofilm biomass of the WT strain increased approximately four-fold between W1 and W3, and 20-fold between W3 and W5, indicating slow biofilm growth during the early phase and accelerated development in the later phase. However, the mutant strain exhibited a 6-fold increase between W1 and W3 and a 5.6-fold increase between W3 and W5, indicating faster biofilm development during the early phase but attenuated biofilm development at the late phase.

In relation to the WT, the mutant strain produced significantly higher quantities of biofilm than the WT strain at W1 and W3 (*p* value = 0.0022), resulting in 1.6-fold and 2.2-fold increases, but was attenuated for biofilm formation at W5, producing significantly lower quantities of biofilm than the WT (*p* < 0.05), yielding a 2.2-fold reduction in biofilm quantities.

#### 2.4.2. Changes in Extracellular K^+^ Concentrations During Biofilm Growth

To determine the role of the K^+^ concentration during biofilm formation, the extracellular K^+^ concentrations in undiluted supernatant fractions of biofilm cultures used for rates of biofilm formation were determined at the different time points, as previously described [21] (Figure 3b, Supplementary Table S4).

The extracellular K^+^ concentrations were 4.435 ± 0.029 mM at W0 for both strains. For both the WT and KT-triple-knockout mutant strains, the K^+^ concentrations were decreased at the three time points during biofilm formation, indicating utilisation of K^+^ by the bacteria during biofilm formation.

In relation to the WT strain, the K^+^ concentrations in the mutant samples were significantly different from those of the WT strain at all time points (*p* values < 0.05) (Figure 3b, Supplementary Table S4), being lower at early and late-biofilm formation phases, indicating increased utilisation during early biofilm formation and the mature biofilm phase. The K^+^ concentrations were higher than the WT at the mid-biofilm formation phase, suggesting attenuation in utilisation at mid-biofilm development.

#### 2.4.3. Changes in Extracellular pH Levels During Biofilm Growth

To determine the effects of pH during biofilm development, the extracellular pH measurements were determined in supernatant samples used for extracellular K^+^ concentration determination, as absolute pH values, at the different time points (Figure 3c, Supplementary Table S5).

The pH levels were 7.2 ± 0.026 at W0, for both strains. For both the WT and the KT-triple-knockout mutant strains, the extracellular pH levels were more alkalinic at W1 and W3 than at W0, but were declining at W5, although still higher than the neutral pH levels.

In relation to the WT strain, the pH levels fluctuated during the different phases of biofilm formation, being higher than the WT strain at early biofilm and mature biofilm, and lower than the WT at mid-biofilm phases (*p* < 0.05) (Figure 3c, Supplementary Table S5).

In summary, the biofilm formation results show that despite higher biofilm formation at early stages of biofilm development, biofilm formation in the mutant strain was attenuated at the final stages. These effects corresponded with lower K^+^ concentrations and elevated pH levels at the early and mid-biofilm development stages, while both measurements decreased at the late stages of biofilm development for both strains.

### 2.5. Macrophage Intracellular Survival

#### 2.5.1. Bacterial Uptake by Macrophages

**Figure 4:**
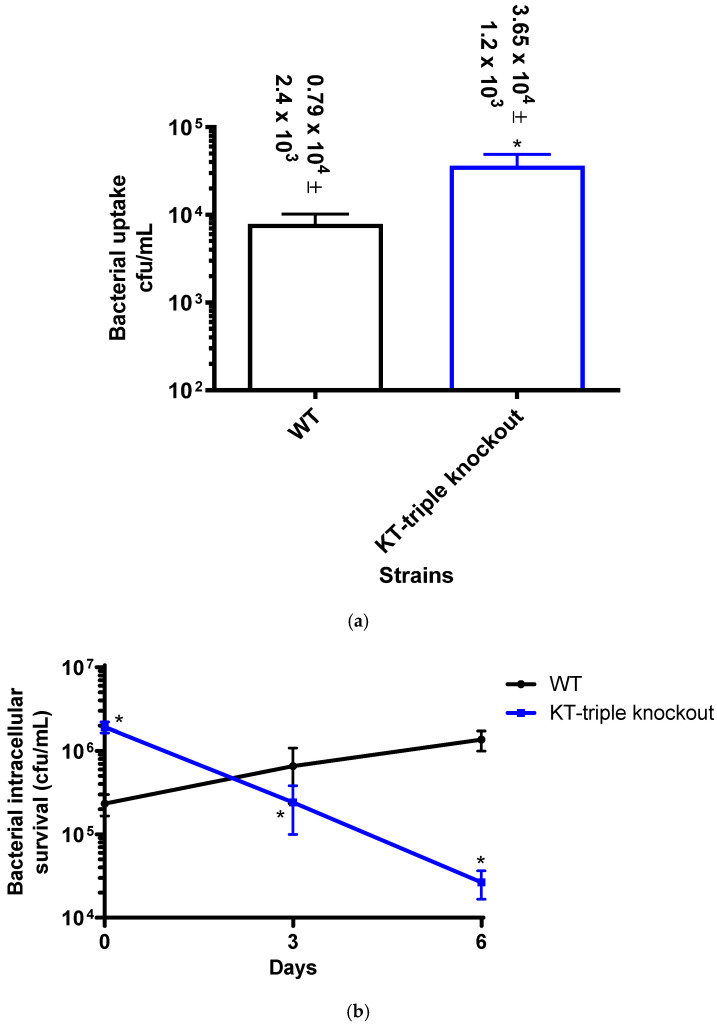
Assays of uptake and intracellular growth of the wild-type (WT) and the Kdp-Trk (KT)-triple-gene knockout mutant strains by human monocyte-derived macrophages. The results are of three experiments performed in duplicate. Statistical differences at *p* values ≤ 0.05 are represented by *, representing a comparison of responses between the WT and the mutant strains. (**a**) Macrophage uptake of the WT and mutant strains of *M. tuberculosis* at one-hour incubation showing a *p* value = 0.0022. (**b**) Intracellular bacterial survival of the two strains at different time points (Supplementary Table S6), showing *p* values of 0.0022, 0.0163 and 0.0022 for days D0, D3 and D6, respectively.

To assess the role of K^+^-uptake transporters in macrophage phagocytosis, the efficiency of bacterial uptake by isolated human blood monocyte-derived macrophages was determined by evaluation of the number of internalised bacteria at one-hour post bacterial infection. The numbers of intracellular bacteria were 0.79 × 10^4^ ± 2.4 × 10^3^ and 3.65 × 10^4^ ± 1.2 × 10^3^ cfu/mL/well, for the WT and mutant strains, respectively (Figure 4a, *p* value = 0.0022), showing 10% and 25% uptake of their inocula, respectively.

#### 2.5.2. Intracellular Growth of Internalised Bacteria

To determine the rates of intracellular growth and survival of each strain, the numbers of intracellular bacteria were determined at D0, D3 and D6 time points post infection, representing the early intracellular survival (18 h: day), intermediate active growth (48 h: 2 days), and late survival phases (110 h: 4.5 days) respectively [34] (Figure 4b, Supplementary Table S6).

Both strains have demonstrated increases in bacterial numbers at D0. The numbers of bacteria have increased from the bacterial internalisation phase to D0 by 30-fold and 53-fold for the WT and the mutant strains, respectively, illustrating intracellular growth. Thereafter, the numbers of intracellular bacteria of the WT strain increased by 3-fold and 2-fold at D3 and D6, respectively, illustrating intracellular growth, while the mutant showed bacterial attenuation, with the intracellular bacterial numbers decreasing by 8-fold and 9-fold at D3 and D6, respectively.

In relation to the WT strain, the intra-macrophage numbers of the mutant strain were significantly higher than those of the WT at D0, demonstrating an 8.25-fold increase (*p* value = 0.0022) while the bacterial numbers decreased by 2.72-fold (*p* value = 0.0163) and 51-fold (*p* value = 0.0022), at D3 and D6, respectively, showing bacterial attenuation.

These macrophage assay results showed that the mutant strain demonstrated higher uptake macrophages, but was attenuated for intracellular growth and survival.

## 3. Discussion

The M. tuberculosis utilise two prominent K^+^-uptake transporters, namely the low-affinity Trk and high-affinity KdpFABC, which differ with respect to properties and functions, for growth and survival in varying conditions of external K^+^ concentration and pH levels [4,12,13,17,41]. The bacteria utilise the Trk system while the KdpFABC is suppressed during growth [10,13]. However, in the absence of the Trk, the KdpFABC is derepressed and used as a backup [8], underscoring its adaptability to growth conditions. During survival in hostile K^+^-limiting, acidic environments, the bacteria upregulate the genes, which encode both transporters [5,8,26,42], both of which are regulated by the KdpDE system [4,17,19,21,29,43].

Although these findings demonstrate the roles of the individual systems in varying environmental conditions, the operational requirement for the coexistence of the Trk and Kdp systems in promoting bacterial growth and survival is not known. This important issue was investigated in the current study, firstly using a homologous recombination procedure to construct a mutant strain of *M. tuberculosis* in which the three K^+^ transport systems, namely Trk, KdpFABC and KdpDE, were inactivated. Secondly, construction of the KT-triple-gene knockout mutant was followed by evaluation of this mutant strain with respect to growth kinetics in planktonic cultures and survival in biofilm and macrophage cultures, relative to the WT strain.

### 3.1. Planktonic Growth

The absence of the two active K^+^-uptake transporters in the KT-triple-knockout mutant strain did not attenuate, but actually increased bacterial growth. The mutated bacteria were able to transport K^+^ inwardly, illustrating the utilisation of an alternative K^+^ transporter for K^+^ uptake and growth. Although not yet fully characterised, the alternative transporter/s may include K^+^ channels encoded by the *M. tuberculosis*, such as the putative transmembrane cation transporter, Rv3200c [30,44], and a conserved hypothetical protein, Rv3237c [25], which are both non-essential for growth, but serve as a backup in the absence of the active transporters.

We have previous showed that mycobacterial utilisation of the low-affinity Trk results in slower growth rates [13]. Furthermore, we showed that in the absence of the Trk, the bacteria derepress the inducible high-affinity KdpFABC, resulting in high K^+^ uptake and ultimately faster growth rates [8]. These previous findings imply that the faster growth rates of the KT-triple-gene-knockout mutant strain observed in the current study are a result of increased K^+^ influx, resulting from the activity of the backup K^+^-uptake transporter(s).

Previous studies from our group and other researchers have demonstrated that *M. tuberculosis* grows optimally at elevated pH levels [2,3,45] by promoting bacterial K^+^ uptake [8,21]. The high K^+^ influx and faster growth rates of the mutant strain in the current study are therefore attributable in all probability to the elevated external pH conditions.

We have previously shown that the activities of the Trk and the KdpFABC systems are optimised by the KdpDE system, showing sluggish bacterial growth in its absence [21]. Other studies have demonstrated their activities and interactions during growth [29,30]. The faster growth rates of the KT-triple-gene knockout mutant in the absence of the KdpDE are also indicative of a regulatory role of the KdpDE in modulating the activities of the backup K^+^ uptake system to slow mycobacterial growth. This contention is supported by the finding in a mouse model of experimental TB demonstrating high virulence of a KdpDE-deletion mutant of the bacterial pathogen, illustrating its role in regulation of bacterial growth [46].

However, in acidic conditions at the late-log phase, mycobacteria slow their growth rates, despite the elevated extracellular K^+^ concentrations, for survival [3,8,11,41]. However, in environments of elevated extracellular pH levels, where the Trk is functional, *M. tuberculosis* utilises both the Trk and Kdp systems [21]. The slow growth rates of the KT-triple-gene knockout mutant strain in acidic, but K^+^ saturated, conditions illustrate the utilisation of the alternative transporter/s by the bacteria for survival in these conditions.

In summary, the absence of the Trk and Kdp systems leads to the utilisation of an inducible backup high-affinity K^+^ uptake transporter(s) for K^+^ uptake and ultimately growth. Similar to the two active K^+^-uptake transporters, Trk and KdpFABC, the functions of this putative alternative backup system may also be regulated by the KdpDE.

### 3.2. Biofilm Cultures

In stressful environments such as low K^+^ concentrations, acidic pH, nutrient limitation and hypoxia, mycobacteria form biofilm for survival by synthesising and surrounding themselves with protective extracellular polymeric substances (EPSs) [32,47]. The bacteria survive by forming aggregate communities, which alter their metabolism and transition to a state of dormancy [37,48]. In the current study, both the WT and the mutant strain were able to form biofilm.

Previous studies have implicated the CeoB protein of the Trk system in biofilm formation by *M. tuberculosis* [25,26]. Although not yet known, the role of the *M. tuberculosis* Kdp system in dormancy observed in low-K^+^ [4,6] and acidic environments [8,11,41] of planktonic cultures suggests a potential role for this K^+^ transporter in biofilm formation. In the current study, the formation of biofilm by the mutant strain illustrates the utilisation of an alternative K^+^-uptake system in biofilm formation, which, similar to planktonic growth, may involve the K^+^ channels.

We also demonstrated that both the WT and the mutant strains produced lower quantities of biofilm at the early and mid-stages of biofilm growth reaching optimum quantities at the late phase of biofilm development. Previous studies have showed that *M. tuberculosis* formed mature biofilm at W5 in vitro [37]. However, biofilm formation of the mutant strain fluctuated, resulting in higher quantities than those of the WT at the early and mid-stages, showing faster rates of biofilm development, but slow rates of biofilm maturation in the late phase.

Regarding the biofilm environments, we demonstrated that the bacteria utilise K^+^ for biofilm formation resulting in K^+^ turnover during biofilm formation. However, the pH levels fluctuated during biofilm formation, being elevated at the development stage (W1 and W3) [49] and decreasing at the mature biofilm stage (W5) [37,38], highlighting a low-K^+^, high-pH environment for biofilm development and a low-K^+^, low-pH condition for mature biofilm formation by *M. tuberculosis*. In the case of the KT-triple-gene knockout, both the early and late stages of biofilm formation were associated with low-K^+^, elevate-pH environments favouring biofilm development but slow biofilm maturation, showing slow pH modulation to acidity by the bacteria, highlighting the limitation of the backup K^+^ transporter in biofilm optimisation. Similar to planktonic growth, the activity of the backup system may be dependent on the regulatory activity of the KdpDE regulating the biofilm development and optimising maturation.

The attenuation of biofilm formation by the mutant strain highlights the vulnerability of the bacteria to external factors. These results show that the active K^+^ transporters may serve as potential drug targets, allowing bacterial accessibility to external factors.

### 3.3. Macrophages

Previous studies demonstrated the role of the Trk transporter during mycobacterial infection of macrophages showing bacterial attenuation in phagocytosis of the CeoBC-deletion mutant strain of *M. tuberculosis* in the bone marrow-derived macrophages [10]. The ability of the mutant strain to infect macrophages shows the utilisation of an alternative K^+^ transporter for infection via phagocytosis.

The aforementioned contention is supported by a previous study, which demonstrated the essentiality of K^+^ for bacterial colonisation [10]. The *M. tuberculosis* phagosome has been shown to be saturated with K^+^ (19–40 mM) [50], with K^+^ concentrations increasing during phagosomal maturation [10]. The high infectious rates of the mutant suggest that the backup transporter has a high affinity for the cation, increasing bacterial K^+^ influx, which, in turn, supports bacterial infection of macrophages. Additionally, the high infectious rates may be as a result of the absence of the KdpDE, which is known to regulate other K^+^ uptake transporters. These results are consistent with the involvement of the active K^+^-uptake transporters in slowing bacterial infectivity rates by slowing phagocytosis.

Following phagocytosis, mycobacteria are exposed to the intracellular anti-mycobacterial effector mechanisms of macrophages, which determine their growth or survival, such as acidic pH, oxidative and nitrosative stressors, and nutrient restriction [11,20,51]. Previous studies have shown the roles of the active transporters in bacterial survival, implicating upregulation of the *ceoB* (Rv2691) [33] gene and the entire *ceoBC* operon in promoting intracellular survival in mouse monocyte-derived macrophages [10], while the *kdpE* gene was upregulated during the mid-stage (48 h post infection) of bacterial infection in macrophages [34].

However, with respect to the intracellular milieu, the WT strain manifested bacterial replication while the mutant strain was attenuated for survival. Failure to survive indicates that the alternative K^+^ transporter utilised by the KT-triple-gene knockout mutant is unable to support mycobacterial survival, despite showing high infectivity rates. These observations underscore the necessity of the operational efficacy of the major mycobacterial K^+^ transporters to modulate growth conditions in macrophages, which are favourable to replication of the bacterial pathogen.

The findings of the current study indicate that under optimal growth conditions, such as in planktonic cultures, bacterial growth is unregulated in the absence of the transporters. In contrast, K^+^ transporters are essential for survival, as their absence leads to reduced bacterial survival in harsh environments in biofilm and macrophages.

These findings may not, however, accurately reflect the situation during host infection with *M. tuberculosis*, in which bacteria are found in macrophages and granuloma lesions, where they interact with the host immune system. Due to different growth conditions between the in vitro and in vivo environments, further investigation is required to evaluate the growth and survival of the KT-triple-gene-knockout of *M. tuberculosis* in natural environments in animal models of infection, specifically the guinea pig model, which leads to formation of granuloma lesions similar to those which develop in humans [52]. Although the present study demonstrates significant differences between the WT and triple-gene-knockout mutant during growth and survival under varying environmental conditions, comparisons between the triple-gene-knockout and the corresponding single- and double-gene-knockout mutants could not be performed, limiting a clear understanding of the individual contributions of each system. In this regard, limited information is available on the roles of the Trk [8,10,13] and KdpDE [21,46] systems, excluding KdpFABC, in planktonic growth, based on single-gene deletion mutants, and no information exists on the contribution of individual mutants to survival in biofilms and/or within macrophages. The lack of characterisation of single mutants represents an important limitation that needs to be addressed in future studies to enable a comprehensive understanding of the specific roles of each transporter in the observed phenotypes. Therefore, these findings constitute a preliminary study.

## 4. Materials and Methods

### 4.1. Materials

#### 4.1.1. Bacterial and Plasmid Strains

The details of all the plasmid and bacterial strains are described in Table 1. The plasmids, pNILRB5 and pGOAL17, were provided by Professor N Stoker and Dr R Balhana (Royal Veterinary College, London, UK) while all other plasmids were constructed in this study.

The bacterial strains included the *Escherichia coli* (*E. coli*) strain DH5α Z-competent™ (Zymo Research, Irvine, CA, USA), purchased from Inqaba Biotecnical Inductries, Pretoria, Gauteng, South Africa and the *M. tuberculosis* strains, the WT (H_37_Rv) American Type Culture Collection (ATCC) 26518 and the KT-double-gene mutant (Δ*kdpDE*-Δ*ceoBC::hyg^R^*), which were used previously [21]. The *E. coli* and *M. tuberculosis* WT strains were used for cloning purposes while the KT-double-gene mutant strain was used for homologous recombination.

#### 4.1.2. Growth Media

The Luria–Bertani broth (LB) and agar (LA) media were used for *E. coli* bacterial culture preparations, while the Middlebrook 7H9 broth supplemented with 10% oleic acid–albumin–dextrose–catalase (OADC), 0.2% glycerol and 0.05% Tween 80, Middlebrook 7H10 agar supplemented with 10% OADC and 0.5% glycerol [39,40] and Sauton’s broth medium [37,39] were used for *M. tuberculosis* culture preparations. The Roswell Park Memorial Institute (RPMI) 1640 tissue culture medium (BioWhittaker, Walkersville, MD, USA) (supplemented with an antibiotic mixture [penicillin: streptomycin: amphotericin B, 0.1: 0.25: 0.1 µg/mL], 5% autologous serum) was used for macrophage culture preparation [53].

#### 4.1.3. Antimicrobial Agents, Enzymes, Chemicals and Reagents

The antibiotics, kanamycin (10 µg/mL), hygromycin (50 µg/mL) and ampicillin (100 µg/mL), purchased from Sigma-Aldrich Chemicals Co, St Louis, MO, USA), were used for the selection of antibiotic-resistant clones. All enzymes (BsaI, BseRI, PacI, T4 DNA polymerase and T4 DNA ligase) (Inqaba biotecnical, Pretoria, South Africa) were used for cloning purposes.

The 5-bromo-4-chloro-3-indolyl-β-D-galactoside (X-gal) (0.24 mg/L) was used for the selection of β-galactosidase gene-expressing clones, while sucrose, at 5% and 2% *w*/*v*, was used for selection of the levansucrase (*sacB*) gene-expressing *E. coli* and *M. tuberculosis* bacterial isolates, respectively. Rubidium-86 (^86^Rb^+^) isotope (PerkinElmer, Johannesburg, South Africa) purchased at the 1 mCi/mL concentration was used as a tracer for K^+^. All other reagents used were purchased from Sigma-Aldrich Chemicals Co., (Sigma-Aldrich, Inc., St Louis, MO, USA), Inqaba biotecnical Industries (Inqaba Biotec, Pretoria, Gauteng, South Africa), Roche (Basel, Switzerland) and Lasec^®^ (Cape Town, Western Cape, South Africa).

### 4.2. Methods

#### 4.2.1. Construction of the KT-Triple-Gene Knockout Mutant Strain

The KT-triple-gene knockout mutant was constructed using homologous recombination following a two-step strategy, as described previously [35].

##### Construction of SDV

The SDV was constructed using the pNILRB5 vector following the LIC procedure, as described [36]. Briefly, T4 polymerase-treated PCR-synthesised, *kdpDF’* and *kdpC′* fragments were sequentially ligated into T4 polymerase-treated BsaI- and BseRI-pNILRB5 vectors to form pRB5*kdpDF’* and pRB5*kdpDFC′* vectors, respectively. Thereafter, a 6359 bp PacI-*lacZ*, *sacB* marker cassette, from pGOAL17, was ligated into the pRB5*kdpDFC′* vector forming the pRB5*kdpDFC*17′ SDV.

##### Construction of the *M. tuberculosis* KT-Triple-Gene Knockout Mutant

The SDV was ultraviolet (UV)-pretreated (5 μg) and electroporated into the *M. tuberculosis* KT (Δ*kdpDE*-Δ*ceoBC::hyg^R^*)-double-knockout mutant strain, followed by isolation of the SCOs and subsequently the DCO, as previously described [35]

#### 4.2.2. PCR Analysis

The PCR analysis was performed to confirm the *kdpFABC* mutation following the procedure, as described previously [36]. DNA extraction from the WT strain was as previously described [21], and the primer sequences for the reaction mixtures were as shown in Table 2 and Table 3. The PCR was carried out in a StepOne Plus thermocycler (Applied Biosystems, Foster City, CA, USA) where the samples were predenatured at 94 °C for 5 min, followed by 35 cycles of amplifications including denaturation at 94 °C for 15 s, annealing at 55 °C for 30 s, and an extension at 68 °C for 75 s, followed by final extension at 68 °C for 5 min and a hold step at 4 °C indefinitely.

#### 4.2.3. WGS Analysis

The SDV and the *M. tuberculosis* mutant were also confirmed using WGS analysis. Multiplexed paired-end libraries (2 × 300 bp) were prepared using the Nextera XT DNA sample preparation kit (Illumina, San Diego, CA, United States) and sequences determined on an Illumina MiSeq platform with 100× coverage at the National Institute of Communicable Diseases (NICD) Sequencing Core Facility, National Health Laboratory Services (NHLS), Johannesburg, Gauteng, South Africa.

#### 4.2.4. Inoculum Preparation

Inocula of the WT and the KT-triple-gene knockout mutant strains were prepared as previously described with minor modifications [21]. Briefly, for each strain, bacterial culture was grown in 7H9 broth to the mid-log phase at 37 °C under aerobic conditions. The cells were harvested by centrifugation at 2851× *g*, and room temperature, for 15 min. The cell pellet was washed twice with phosphate-buffered saline (PBS: pH 7.4) and resuspended in 7H9 broth, followed by OD determination to 1.2 at 540 nm. The numbers of bacteria in each inoculum were determined by a plating method, as previously described [39].

#### 4.2.5. Planktonic Assays

##### Preparation of Planktonic Cultures

The planktonic cultures were prepared as previously described [21]. Approximately 10^5^ cfu/mL of cells were inoculated into 2 mL 7H9 broth in 24-well tissue culture plates (Greiner Bio-One GmbH, Frickenhausen, Germany) at 37 °C for 15 days in the dark under aerobic conditions. Bacterial growth was determined spectrophotometrically by OD measurement at 540 nm.

The planktonic cultures were used for the determination of ^86^Rb^+^-uptake efficiencies, rates of bacterial planktonic growth and measurement of extracellular pH levels.

##### Rates of Planktonic Growth

The cultures were sampled every three-day time points beginning at D0 to D15, and growth was determined spectrophotometrically by OD measurements at a wavelength of 540 nm. The rates of growth of each strain were determined as the level of growth at each time point for each strain. The rates of growth in the mutant were compared with those of the WT strain.

##### Rubidium Uptake

Rubidium (^86^Rb^+^) was used as a surrogate tracer for K^+^ for determination of the K^+^-uptake efficiencies of the WT and the mutant strains. Briefly, following culture growth for 15 days, the cells were harvested and resuspended to ca. 10^6^ cfu/mL in K^+^-, nitrogen-free buffer (KONO) containing 2 mCi/L ^86^Rb^+^ and uptake of the radioisotope determined as absolute cpm. The ^86^Rb^+^-uptake efficiency of the mutant strain was compared to that of the WT.

##### pH Determination of Planktonic Cultures

The cultures used for rates of growth determination were used for measurement of extracellular pH levels. The culture supernatants were harvested by centrifugation (2851× *g*, 15 min), and the pH levels were measured directly in the undiluted samples using pH 4 and 7 as references in the Janeway pH meter 3510 (Lasec, Johannesburg, South Africa). The results were recorded as absolute pH values.

#### 4.2.6. Biofilm Assays

##### Preparation of Biofilm Cultures

The biofilm-forming cultures were prepared as previously described [39]. Approximately 10^5^ cfu/mL of cells were inoculated into 2 mL Sauton’s broth medium, in 24-well tissue culture plates (Greiner Bio-One GmbH, Frickenhausen, Germany). The plates were incubated at 37 °C in the presence of 5% CO_2_ in the dark, under anaerobic conditions for five weeks.

The amounts of biofilm biomass in the cultures were quantitated using a CV-based staining procedure as described [39] with minor modifications. The supernatants, containing planktonic cells in the biofilm-forming cultures, were removed, and the residual biomass in the wells was washed once with 1 mL distilled water (dH_2_O) and air-dried. The residual matrix was stained with 1 mL of 1% CV solution and incubated for 30 min at room temperature followed by three washes with 1 mL dH_2_O to remove the unbound CV dye, and air-dried. The biofilm-associated CV was extracted with 1 mL of 70% ethanol, followed by 10-fold dilution and measurement of OD at 570 nm using a Spectronic Helios UV-visible (Vis) spectrophotometer (Merck, Burlington, MA, USA).

The cultures were used for the determination of rates of biofilm formation, extracellular K^+^ concentrations and pH levels.

##### Rates of Biofilm Growth

The biofilm quantities were determined at W1, W3 and W5, for each strain, and the rates of biofilm formation were determined by measuring the amount of biofilm formed at each time point. The rates of biofilm formation in the mutant strain were compared to those of the WT at each time point.

##### Measurement of Extracellular pH Levels and K^+^ Concentrations

The supernatants were harvested from the biofilm cultures, centrifuged and decontaminated as described for planktonic cultures. Thereafter, the pH levels were measured as described for planktonic cultures. The K^+^ concentrations were measured by indirect potentiometry utilising a K^+^-selective electrode in conjunction with a Na^+^-reference electrode using the Beckman Coulter Synchron LX 20 System (Beckman Coulter, Ireland Inc., Gateway, Ireland) in the undiluted samples [8].

#### 4.2.7. Macrophage Assays

##### Preparation of Macrophages

Macrophages were prepared from human blood-derived monocytes, as previously described [53]. The monocytes were isolated from venous blood, drawn from healthy (no history of TB disease, no medication and no smoking), adult (>20 years), human volunteers after permission was granted by the Main Research Ethics Committee of the Faculty of Health Sciences, University of Pretoria, and acquisition of consent from participants. Heparinised blood was layered on atop Histopaque (Sigma) and centrifuged (25 min 400× *g*, room temperature). The mononuclear leukocyte (MNL) fraction at the plasma/Histopaque-1077 interface was harvested and washed with PBS (pH 7.4). The contaminating erythrocytes were removed by hypotonic lysis, and the cells were resuspended in sterile Hanks’ Balanced Salt Solution (HBSS, indicator-free, containing 1.25 mM calcium chloride (CaCl_2_) [pH 7.4], Highveld Biological, Johannesburg, South Africa) and analysed for total T lymphocytes, monocytes, granulocytes, and B lymphocytes using the fluorochrome-labelled monoclonal antibodies (all Beckman Coulter, Miami, FL, USA): anti-CD3 (FITC: fluorescein isothiocyanate), anti-CD14 (PE: phycoerythrin), anti-CD15 (FITC), and anti-CD19 (PE), respectively using a flow cytometry (FC)500 flow cytometer (Beckman Coulter, USA) using CXP software. The monocytes were separated from other MNL cells by differential adherence to plastic onto the surface of 75 cm^3^ tissue culture flasks for 2 h at 37 °C in the presence of 5% CO_2_. The adherent cells were propagated into macrophages in the tissue culture medium RPMI 1640 (Bio Whittaker, Walkersville, MD) supplemented with antibiotic mixture (penicillin/streptomycin/amphotericin B, 0.1/0.25/0.1 mg/mL) and 5% autologous serum for seven days at 37 °C. The cells were treated with PBS containing Ca^2+^-chelating ethylene glycol-bis (2-aminoethylene)-N,N,N,N-tetracetic acid (EGTA, 2 mM, final) and scraped from the flask, resuspended and analysed for purity of the isolated macrophages and their viability using the FC500 flow cytometer.

##### Macrophage Infection with *M. tuberculosis*

Approximately 1 × 10^5^ macrophage suspension (200 µL) was plated in 48-well tissue culture plate wells followed by treatment with 10 mM CaCl_2_, for two hours to aid macrophage adherence. Thereafter, the cells were grown in antibiotic-free RPMI 1640/5% autologous serum (500 µL) at 37 °C/5% CO_2_ for 24 h. The adherent cells in the wells were washed once with 500 µL pre-warmed PBS, and approximately 1 x 10^6^ cfu/mL (200 µL) in antibiotic-free RPMI 1640/5% autologous serum was added to achieve a multiple of infection (MOI) of 10:1, bacteria: macrophages, followed by incubation of the culture plate at 37 °C/5% CO_2._ The wells were washed once in PBS to remove the non-internalised bacteria followed by incubation of the infected macrophages in a volume of antibiotic-free RPMI 1640/5% autologous serum (500 µL) at 37 °C in the presence of 5% CO_2_ for different time points for bacterial growth and survival.

##### Measurement of Intracellular Growth and Survival

Intracellular bacterial growth was determined by treating the wells with 0.2% sodium dodecyl sulphate (SDS) (100 µL) for 20 min, and thereafter, serial dilutions of the contents of the wells were made and plated on 7H10 agar medium for colony development. The numbers of colonies were counted and used for determination of the number of bacteria using dilution theory [40,53].

##### Bacterial Uptake Determination

For bacterial uptake determinations, intracellular bacteria were determined at one hour following bacterial infection.

##### Rates of Intracellular Survival

Following bacterial infection, the rates of intracellular bacterial survival were determined by enumeration of the number of intracellular surviving bacteria at different time points, which included 18–24 h, referred to as D0, D3 and D6, representing early intracellular survival, intermediate active growth, and late survival phases respectively. The rates of growth between the mutant and the WT strains were compared at all time points.

### 4.3. Statistical Analysis and Presentation of Data

Data was analysed using Graph Pad statistical package Instant 3 Programme (GraphPad Software, San Diego, CA, USA), and the results of each series of experiments were presented as means ± standard deviations. Statistical differences between the WT and mutant strains were determined by a *p* value < 0.05, taken as being statistically significant, using an unpaired Student *t*-test/Mann–Whitney U-test.

## 5. Conclusions

The findings of the current study using the KT-triple-gene knockout mutant strain of *M. tuberculosis* appear to implicate the existence of alternative K^+^ uptake mechanisms utilised by *M. tuberculosis*. In this context, K^+^-uptake membrane channels have been described in *M. tuberculosis*, and further research involving characterisation and possible involvement of these ion channels in bacterial growth may enable the identification of additional drug targets.

Furthermore, the findings of the current study demonstrate that the major K^+^ transporters of *M. tuberculosis*, Trk and Kdp K^+^ transporters, harmonise in regulating the virulence of the pathogen, with their involvement being evident during bacterial growth in planktonic conditions, as well as survival in biofilm development/maturation and macrophages, underscoring their potential as novel drug targets.

Finally, dual inactivation of the Trk and Kdp systems was found to render the KT-triple-gene-knockout mutant more susceptible to phagocytosis and intracellular killing by isolated human blood monocyte/macrophages. The high virulence and infectious rates of the mutant strain in the setting of attenuation of intracellular growth and survival is indicative of the drug potential of the KT-triple-mutant strain of *M. tuberculosis.*

## Figures and Tables

**Table 1:** Bacterial strains and plasmids used and constructed in this study.

Bacterial and Plasmid Strains	Feature or Genotype	Source
pNILRB5	*kan^R^*, *oriE*, *sacB*, *lacZ*, *PacI* site, *BsaI* and *BseRI* LIC sites	[36]
pRB5*kdpDF′*	*kan^R^*, *oriE*, *sacB*, *kdpDF*, *BseRI* LIC site	This study
pRB5*kdpDFC′*	*kan^R^*, *oriE*, *kdpDF*, *kdpC*, *PacI site*	This study
pGOAL17	*PacI* cassette (*P_Ag85_-lacZ P_hsp_*-*sacB*), *amp*	[35]
pRB5*kdpDFC17′*	pRB5*kdpDFC*′ with *PacI* cassette (*P_Ag85_-lacZ P_hsp_*-*sacB*)	This study
DH5α−*E. coli*	F^−^φ80dlacZΔM15 Δ(lacZYA-argF) U169 deoR, recA1 endA1 hsdR17 (r_k_^−^m_k_^+^ phoA supE44 λ^−^ thi-1 gyrA96 relA1)	[21]
WT H_37_Rv, American Type Culture Collection (ATCC) 26518	All genes encoded	ATCC reference strain
KdpDE-Trk-double gene knockout	Δ*kdpDE*, Δ*ceoBC::hyg^R^*	[21]
Kdp-Trk-triple-gene knockout	Δ*kdpDE*, Δ*kdpFABC*, Δ*ceoBC::hyg^R^*	This study

ATCC, American Type Culture Collection; WT, wild-type.

**Table 2:** Oligonucleotide sequences for the PCR-synthesised inserts for SDV construction.

Primers (bp)	Target	Fragment Size
Forward 5′TACTTCCAATCCATGGCCACGGATAACGTGAACC3′ (34) Reverse 5′TATCCACCTTTACTGCGATGTTGTCGACCGTAGT3′ (34)	*kdpDF*	1164
Forward 5′TATCCACCCTTACTGCGTGCTCAGGCTGAACCTC3′ (34) Reverse 5′TACTTCCAATCCATGGCGCCTACCAGGTTGACAG3′ (34)	*kdpC-trcS*	1143

**Table 3:** Oligonucleotide sequences for PCR analysis for the characterisation of the mutant clone.

Primers (bp)	Fragment Size (bp)	Fragment Size (bp)
	Mutant	Wild-Type
Forward 5′CGGGGAAACAACAGTCGAACT3′ (21) Reverse 5′GCGACTGACATTCCGATC3′ (18)	No fragment	1041
Forward 5′CGGGGAAACAACAGTCGAACT3′ (21) Reverse 5′CCTGGTCATCAACGCGGTG3′ (19)	868	5014

## Data Availability

All the datasets generated for this study are included in the article and Supplementary Materials.

## Supplementary Materials

The following supporting information can be downloaded at: https://www.mdpi.com/article/10.3390/ijms27093962/s1.

## Abbreviations

The following abbreviations are used in the manuscript:

ATCC	American Type Culture Collection
ATPase	Adenosine triphosphatase
CaCl_2_	Calcium chloride
Cfu/mL	Colony-forming units per millilitre
Cpm	Count per minute
CV	Crystal violet
D	Day
DCO	Double cross-over
dH_2_O	Distilled water
DNA	Deoxyribonucleic acid
*E. coli*	*Escherichia coli*
EGTA	Ethylene glycol-bis (2-aminoethylene)-N,N,N,N-tetracetic acid
EPS	Extracellular polymeric substance
FC	Flow cytometry
FITC	Fluorescein isothiocyanate
H^+^	Hydrogen ion, proton
K^+^	Potassium
KONO	Potassium- and nitrogen-free
KT-double	KdpDE-Trk-double mutant
KT-triple	Kdp-Trk-triple-knockout mutant
LA	Luria–Bertani agar
LB	Luria–Bertani broth
LIC	Ligase-independent cloning
*M. tuberculosis*	*Mycobacterium tuberculosis*
MNL	Mononuclear leukocyte
MOI	Multiple of infection
NAD^+^	Nicotinamide adenine dinucleotide
NHLS	National Health Laboratory Services
NICD	National Institute of Communicable Diseases
OADC	Oleic acid-albumin-dextrose-catalase
OD	Optical density
P-type, ATPase	Phosphorylated-type ATPase
PBS	Phosphate-buffered saline
PCR	Polymerase chain reaction
PE	Phycoerythrin
RPMI	Roswell Park Memorial Institute
^86^Rb^+^	Rubidium-86
SCO	Single cross-over
SDS	Sodium dodecyl sulphate
SDV	Suicide-delivery vector
TCS	Two-component system
UV	Ultraviolet
Vis	Visible
W	Week
WGS	Whole-genome sequencing
WT	Wild-type
X-gal	5-bromo-4-chloro-3-indolyl-β-D-galactoside
