# Supplementary material for: The Potassium-Uptake Systems, Trk and Kdp, Coordinately Contribute to Growth Regulation and Survival of M. tuberculosis in Ion-Depleted and Acidic Environments
Source: Int J Mol Sci. 2026 Apr 29;27(9):3962. doi: 10.3390/ijms27093962 (PMC13163235; doi:10.3390/ijms27093962)
Supplement: Supplementary file 1 [file ijms-27-03962-s001.zip › ijms-4152794-supplementary.pdf]

List of Supplementary materials

List of supplementary figures

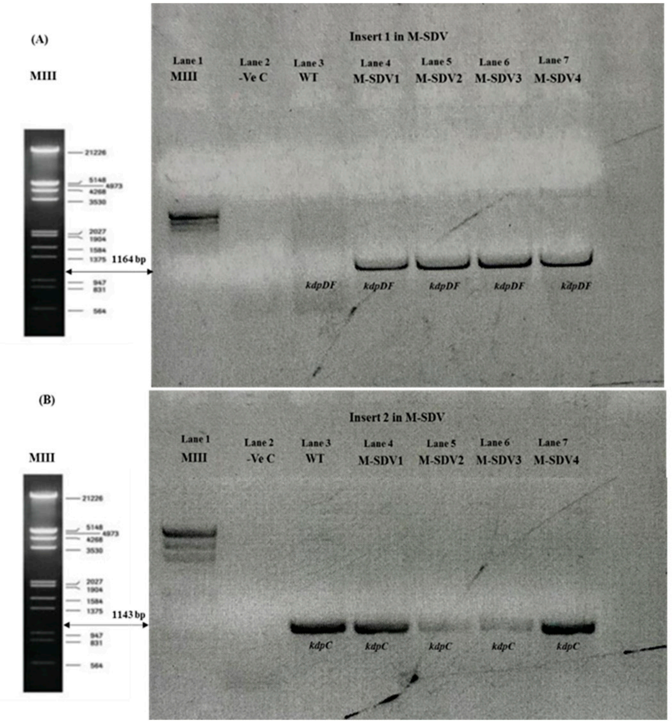

a)

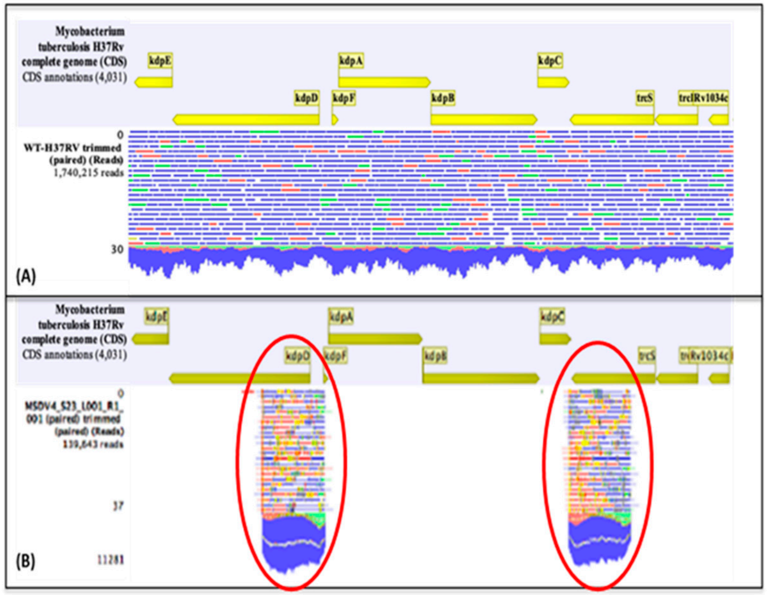

b)

Supplementary Figure S1. The construction of the suicide delivery vector (SDV), *pRB5kdpDFC17`*. a) The SDV map shown by amplification of the 1164-bp *kdpDF* (A) and 1143-bp *kdpC* (B) inserts at the BsaI and BseRI ligase-independent cloning (LIC) sites of the pNILRB5 vector by polymerase chain reaction (PCR) analysis. For both gel maps representing the inserts, the samples in lanes 1-7 represent molecular marker III, no deoxyribonucleic acid (DNA) template, wild-type (WT) $H_{37}Rv$ strain, and SDV1 - 4 respectively. b) The SDV map of SDV4 (B), shown by the presence of the *kdpDF* and *kdpC* reads at the flanking sites of the *M. tuberculosis kdpFABC* operon (A) by whole genome sequencing (WGS) analysis.

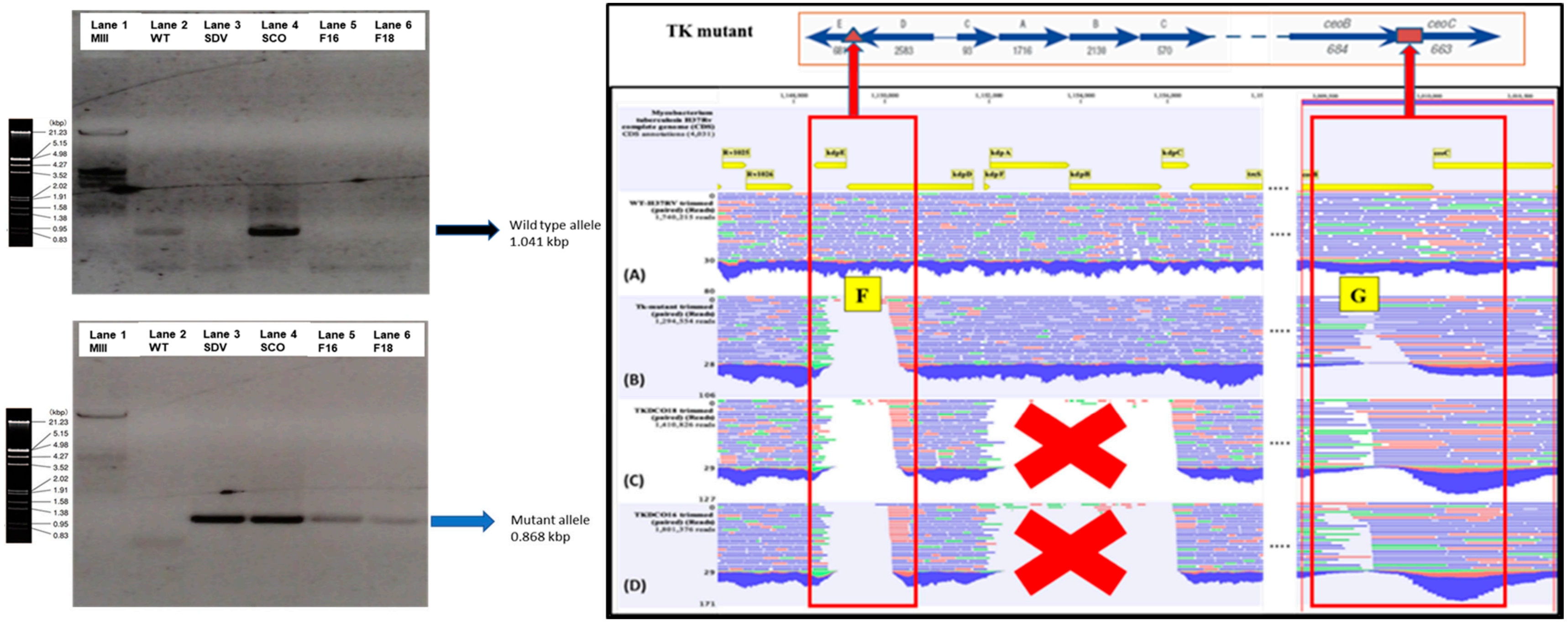

a) b)

Supplementary Figure S2: Construction of the Kdp-Trk (KT: *ΔkdpDE-ΔFABC-ΔceoBC*)-triple gene knockout mutant by homologous recombination. a) Confirmation of the *kdpDFC* mutation by polymerase chain reaction (PCR) analysis. The top and bottom panels of samples represent PCR amplification of the wild-type (WT) and mutated alleles, respectively. The samples in lanes 1 - 6 represent molecular weight marker III, no deoxyribonucleic acid (DNA) template, WT (1041 bp, top panel), suicide delivery vector (SDV: 868-bp, bottom panel), single crossover (SCO: 1041-bp and 868-bp, both panels), and F16 and F18 (mutants: 868-bp, bottom). b) the whole genome sequence (WGS) analysis showing deletion of the *kdpFABC* allele (red crosses), the *kdpDE* and *ceoBC* alleles (red rectangles). The WT and KT-double knockout are represented by A and B, respectively while the KT-triple knockout strain clones are shown by C and D.

List of Supplementary tables

Planktonic cultures

Supplementary Table S1: Rates of planktonic growth (OD at 540 nm)

**Day**	**WT**	**KT-triple gene mutant**	**P value (between strains)**
**0**	0.001 ± 0.0001	0.001 ± 0.0001	NA
**3**	0.005 ± 0.004	0.029 ± 0.015	0.0022
**6**	0.044 ± 0.024	0.247 ± 0.013	0.0022
**9**	0.644 ± 0.090	1.381 ± 0.057	0.0022
**12**	2.034 ± 0.126	2.377 ± 0.009	0.0649
**15**	2.468 ± 0.064	2.438 ± 0.032	0.59

KT-triple, Kdp-Trk; NA, not applicable; WT, wild-type

Supplementary Table S2: pH levels during planktonic growth

**Day**	**WT**	**KT-triple gene mutant**	**P value (between strains)**
**0**	6.7 ± 0.02	6.7 ± 0.02	NA
**3**	6.648 ± 0.006	6.663 + 0.011	0.0163
**6**	6.664 ± 0.003	6.674 + 0.007	0.0104
**9**	6.701 ± 0.009	6.838 + 0.007	0.0022
**12**	6.537 ± 0.009	6.385 + 0.008	0.0022
**15**	6.382 ± 0.009	6.403 + 0.013	0.0304

KT-triple, Kdp-Trk; NA, not applicable; WT, wild-type

Biofilm cultures

Supplementary Table S3: Rates of biofilm growth (OD at 570 nm)

**Week**	**WT**	**KT-triple gene mutant**	**P value (between strains)**
**W1**	0.173 ± 0.02	0.282 ± 0.008	0.0022
**W3**	0.750 ± 0.014	1.672 ± 0.014	0.0022
**W5**	20.31 ± 0.05	9.347 ± 0.025	0.0022

KT-triple, Kdp-Trk; NA, not applicable; WT, wild-type

Supplementary Table S4: External potassium (K^+) concentration during biofilm growth (mM)

Week	WT	KT-triple gene mutant	P value (between strains)
**W0**	4.435 ± 0.029	4.435 ± 0.029	NA
**W1**	4.275 ± 0.02	4.22 ± 0.01	0.0022
**W3**	4.102 ± 0.043	4.172 ± 0.017	0.0082
**W5**	3.825 ± 0.14	3.655 ± 0.016	0.016

KT-triple, Kdp-Trk; NA, not applicable; WT, wild-type

Supplementary Table S5: Extracellular pH levels during biofilm formation

Week	WT	KT-triple gene mutant	P value (between strains)
**W0**	7.228 ± 0.026	7.228 ± 0.026	NA
**W1**	7.196 ± 0.068	7.244 ± 0.069	0.0022
**W3**	7.405 ± 0.046	7.37 ± 0.045	0.0022
**W5**	7.228 ± 0.019	7.261 ± 0.014	0.0022

KT-triple, Kdp-Trk; NA, not applicable; WT, wild-type

Macrophage assays

Supplementary Table S6: Intracellular growth in macrophages (cfu/mL/well)

Day	WT cfu/mL/well	KT-triple gene mutant cfu/mL/well	P value (between strains)
**D0**	$2.34 \times 10^5 \pm 6.7 \times 10^4$	$1.93 \times 10^6 \pm 2.9 \times 10^5$	0.0022
**D3**	$6.6 \times 10^5 + 4.25 \times 10^5$	$2.42 \times 10^5 \pm 1.4 \times 10^5$	0.0163
**D6**	$1.37 \times 10^6 \pm 3.7 \times 10^5$	$2.67 \times 10^4 \pm 9.9 \times 10^3$	0.0022

KT-triple, Kdp-Trk; NA, not applicable; WT, wild-type
